# Racial Differences in Androgen Receptor (AR) and AR Splice Variants (AR-SVs) Expression in Treatment-Naïve Androgen-Dependent Prostate Cancer

**DOI:** 10.3390/biomedicines11030648

**Published:** 2023-02-21

**Authors:** Farhan Khan, Obianuju Mercy Anelo, Qandeel Sadiq, Wendy Effah, Gary Price, Daniel L. Johnson, Suriyan Ponnusamy, Brandy Grimes, Michelle L. Morrison, Jay H. Fowke, D. Neil Hayes, Ramesh Narayanan

**Affiliations:** 1 Department of Pathology, Methodist Hospital, Memphis, TN 38104, USA; 2 Department of Pathology, University of Tennessee Health Science Center, Memphis, TN 38163, USA; 3 Department of Medicine, Division of Hematology and Oncology, University of Tennessee Health Science Center, Cancer Research Building 19, S. Manassas, Room 120 Memphis, Memphis, TN 38103, USA; 4 Molecular Bioinformatics Core, University of Tennessee Health Science Center, Memphis, TN 38103, USA; 5 West Cancer Center, Memphis, TN 38138, USA; 6 Biorepository Core, UTHSC Center for Cancer Research, Memphis, TN 38103, USA; 7 Department of Preventive Medicine, University of Tennessee Health Science Center, Memphis, TN 38163, USA; 8 UTHSC Center for Cancer Research, Memphis, TN 38103, USA

**Keywords:** androgen receptor (AR), AR splice variants (AR-SV), prostate cancer, castration-resistant prostate cancer (CRPC), race, African American (AA), Caucasian American (CA)

## Abstract

Androgen receptor splice variants (AR-SVs) contribute to the aggressive growth of castration-resistant prostate cancer (CRPC). AR-SVs, including AR-V7, are expressed in ~30% of CRPC, but minimally in treatment-naïve primary prostate cancer (PCa). Compared to Caucasian American (CA) men, African American (AA) men are more likely to be diagnosed with aggressive/potentially lethal PCa and have shorter disease-free survival. Expression of a truncated AR in an aggressively growing patient-derived xenograft developed with a primary PCa specimen from an AA patient led us to hypothesize that the expression of AR-SVs could be an indicator of aggressive growth both in PCa progression and at the CRPC stage in AA men. Tissue microarrays (TMAs) were created from formalin-fixed paraffin-embedded (FFPE) prostatectomy tumor blocks from 118 AA and 115 CA treatment-naïve PCa patients. TMAs were stained with AR-V7-speicifc antibody and with antibodies binding to the N-terminus domain (NTD) and ligand-binding domain (LBD) of the AR. Since over 20 AR-SVs have been identified, and most AR-SVs do not as yet have a specific antibody, we considered a 2.0-fold or greater difference in the NTD vs. LBD staining as indication of potential AR-SV expression. Two AA, but no CA, patient tumors stained positively for AR-V7. AR staining with NTD and LBD antibodies was robust in most patients, with 21% of patients staining at least 2-fold more for NTD than LBD, indicating that AR-SVs other than AR-V7 are expressed in primary treatment-naïve PCa. About 24% of the patients were AR-negative, and race differences in AR expression were not statistically significant. These results indicate that AR-SVs are not restricted to CRPC, but also are expressed in primary PCa at higher rate than previously reported. Future investigation of the relative expression of NTD vs. LBD AR-SVs could guide the use of newly developed treatments targeting the NTD earlier in the treatment paradigm.

## 1. Introduction

Approximately 270,000 men in the United States were diagnosed with prostate cancer (PCa) and 34,000 died of PCa in 2022 [1]. Globally, the number of PCa survivors is projected to increase to over 4.5 million by 2026 [2]. Current therapeutic strategies for castration-resistant prostate cancer (CRPC) include androgen receptor (AR) antagonists and a CYP17A1 inhibitor [3,4,5]. Although these drugs extend progression-free survival (PFS), approximately 30% of tumors do not respond to these therapies, and patients who initially respond to these therapies develop resistance shortly after treatment initiation [6]. One of the primary reasons for treatment failure and CRPC relapse is the expression of AR splice variants (AR-SVs) that lack the ligand binding domain (LBD) and are constitutively active [7,8,9]. AR-SVs contribute to CRPC aggressive phenotype, shorter PFS, and failure to respond to enzalutamide or abiraterone [7,10,11,12,13,14]. With a subset of primary PCa not responding to treatment and the reminder developing treatment resistance over time, AR-SV expression is a potential escape mechanism to androgen independency, treatment resistance, and disease progression [15]. 

**Figure 1:**
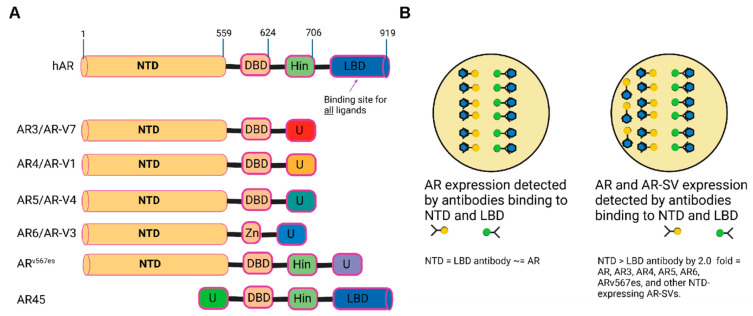
(**A**) AR and AR-SV structure (created using Biorender.com, accessed on 10 December 2022). These are representatives of the 20 AR-SVs clinically detected. (**B**) Diagram representing the method used to detect AR and AR-SVs.

Studies have shown that AR-SVs are expressed in CRPC, but minimally in primary PCa [16,17,18]. Several studies with CRPC specimens and preclinical models identified multiple AR-SVs (Figure 1A) and found that the AR-SVs contribute to cancer relapse after radical prostatectomy [16]. Approximately 20 AR-SVs have been identified preclinically and clinically [10]. Drugs including enzalutamide and abiraterone target the androgen pathway to inhibit PCa progression. However, the treatments fail after an initial period of response, and AR-SVs contribute to the treatment failure [6,19]. For example, AR-V7, a commonly identified AR-SV, in clinical specimens correlated with a unique 59 gene signature in CRPC that corresponded to shorter survival times and resistance to treatments [19]. However, there are many known AR-SVs, and expression levels vary from 30 to 70% in CRPC patients [6,13,19]. 

Advanced CRPC patients treated with enzalutamide and abiraterone expressed AR-V7 at 39% and 19%, respectively, and AR-V7-positive patients had statistically significant lower PSA response rates [6]. Several mechanisms including gene rearrangement and alternate splicing through splicing factors have been attributed to the development of AR-SVs [9,20,21]. Since the AR NTD is the main coactivator interacting surface, the expression of this region in the AR-SVs makes AR-SVs constitutively active and allows them to retain the majority of their activity. Clinically, AR-V7 is detected in both the prostate tissue and in the circulating tumor cells using both at the transcript and protein levels [22]. AR-V7 expression varies widely in hormone-sensitive PCa between studies, ranging from low single-digit percents to over 90% [19,23].

To our knowledge, it is currently unknown whether AR-SV expression levels or patterns differ with race, and no large patient cohorts have been analyzed for a comprehensive expression of AR-SVs in treatment-naïve primary PCa. Compared to Caucasian American men (CA), African American (AA) men have a 63% higher overall PCa incidence. These patients are more likely to be diagnosed with aggressive PCa [24], are 2.44-fold more likely to die from PCa [25], and have shorter disease-free survival [26]. PSA levels remain higher in healthy AA men compared to CA men even after adjusting for age, BMI, and insurance [27,28]. High-grade prostatic intraepithelial neoplasia is more prevalent in younger AA men than age-matched CA men, suggesting an accelerated progression prior to diagnosis [29,30,31]. A previous analysis of men undergoing prostate biopsy that adjusted for clinical and demographic differences found that AA men were 50% more likely to be diagnosed with PCa and 84% more likely to have high-grade PCa than CA men [30]. Among men undergoing radical prostatectomy, AA men were 28% more likely to have a recurrence than CAs [32]. While differential healthcare access may contribute to racial disparities in PCa detection and treatment patterns, when compared to CAs, AA men have higher PSA levels, are diagnosed at a higher grade, show higher tumor expression of adverse molecular markers, and have a higher risk of PCa progression after surgery.

Further characterizing AR-SVs in diverse clinical populations could provide new treatment approaches. In the last decade, several groups have identified molecules that bind to the N-terminus domain (NTD) and inhibit and/or degrade AR and AR-SVs [33,34,35,36]. Considering that the NTD is conserved, and all AR-SVs express the NTD, targeting the NTD would be more effective than a receptor–ligand approach through targeting the LBD. Evaluating the expression of AR-SVs in primary PCa in AA and CA men will determine whether the expression of AR-SVs contributes to development of aggressive PCa or CRPC in AA patients. In this case, targeted clinical assays would be developed, such that existing or new drugs may be administered earlier in the treatment paradigm to improve patient outcomes and prognosis. 

In this study, we evaluated the expression of AR and AR-SVs in treatment-naïve primary PCa from AA and CA men in the mid-south USA. Only two primary prostatectomy PCa tumors stained for AR-V7, both in AA men. However, 20% of the patient specimens stained for NTD at a rate 2.0-fold higher than LBD, indicating AR-SVs other than AR-V7 may serve as potential targets in primary PCa.

## 2. Materials and Methods

AR NTD antibody was procured from Millipore, Burlington, MA, USA (06-680-MI), AR-LBD antibody C19 was from Sigma, St. Louis, MO, USA (SAB5500007), and AR-V7 antibody was obtained from Abcam, Cambridge, UK (ab198394). 

### 2.1. TMA Creation and Staining

Patient specimens were collected under an UTHSC Institutional Review Board (IRB)-approved protocol. Patient details were redacted before the clinical information was released to the researchers. The patient specimens that were collected and stored since 1990 were obtained from the UTHSC center for cancer research biorepository core. Each patient specimen had multiple blocks, and sections were made from each block and sent to the pathologist (FK) for marking the tumor areas. Cores from identified sections were obtained from each block and TMAs were created with a total of 233 specimens (each TMA containing 30 specimens). Twelve specimens showed insufficient tumors and were excluded from the study. Staining was optimized using LNCaP and 22RV1 cells that expressed AR and AR and AR-SV, respectively, with selected PCa slides. Immunohistochemistry protocol was optimized using an automated processor (Leica, Bond III). Cases (*n* = 221) were evaluated for staining with AR NTD and LBD antibodies and 192 cases for AR-V7-binding antibody. We used three AR NTD antibodies (Cell Signaling D6F11) and AR-441 (in addition to PG-21) to optimize staining and chose to use PG-21 antibody for the staining of TMAs. Similarly, the AR-V7 Abcam antibody was compared with RevmAb AR-V7 antibody and chose to continue with the Abcam antibody. Results were scored by two independent pathologists for intensity of staining (0–3) and the number of cells (0–100), and H score was calculated ranging between 0 and 300. 

### 2.2. Statistical Analysis

Fisher’s exact test was used for an analysis of contingency between the African American and Caucasian population with possible splice variants. A similar test was performed on the samples with no staining present. Finally, a Welsh’s *t* test was performed on all samples comparing the fold change in NTD vs. LBD staining. 

### 2.3. Patient-Derived Xenograft

Animal experiments were performed under an UTHSC Institutional Animal Care and Use Committee (IACUC)-approved protocol. Animals were maintained in a 12 h light:dark cycle and were provided with water and food ad libitum. Patient specimens collected from surgical suites in RPMI medium supplemented with penicillin:streptomycin and fungizone were fragmented using collagenase and implanted subcutaneously in male NSG mice.

### 2.4. Western Blot

Tumor tissue from PDX was minced and protein extracted by three freeze thaw cycles in a lysis buffer that contained protease and phosphatase inhibitors. Western blot was performed according to a method previous published [36].

## 3. Results

Since AR-V7 is the only AR-SV with a clinically validated assay, there is a higher chance that the expression of other AR-SVs could be overlooked. Hence, the actual percent of AR-SVs expressed in primary PCa or in CRPC is underestimated. We expected that the approach shown in Figure 1B could provide clarity on the percent of PCa expressing AR-SVs. Two antibodies, one binding to the NTD and one to the LBD, were optimized using LNCaP and 22RV1 cells. LNCaP cell line expresses AR, while 22RV1 expresses AR and AR-SV. LNCaP produced a ratio of 1:1 with the antibodies, while 22RV1 provided a ratio of greater than 2, suggesting that this approach can distinguish between specimens expressing AR only or AR and AR-SVs. We used a cut-off of 2.0 to classify a specimen as AR-positive or AR- and AR-SV-positive. This is similar to the approach adopted earlier [37].

### 3.1. Patient Characteristics and Demographics

**Figure 2:**
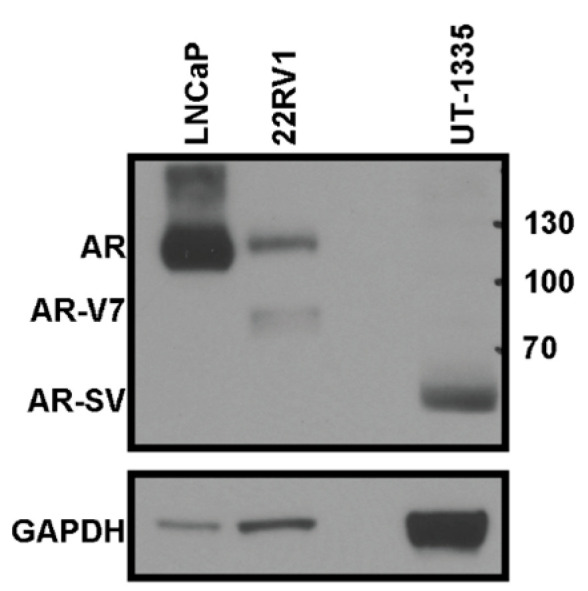
Expression of truncated AR in patient-derived xenograft (PDX). Prostatectomy specimen from an African American man was implanted subcutaneously in NOD-SCID Gamma (NSG) mice. Mice were euthanized and protein was extracted from the PDX, and Western blot for AR was performed with an antibody that binds to the NTD and for GAPDH.

A prostatectomy PCa specimen (UT-1335) from an AA patient aggressively grew from implantation to 2000 mm [3] in under 40 days. Protein was extracted from the specimen and Western blot for AR was performed with an antibody directed to the NTD of AR. Interestingly, the antibody detected a truncated band at ~65 KDa (Figure 2). This led to the assumption that primary treatment-naïve PCa from AA patients could potentially express AR-SVs, and that could potentially contribute to aggressive phenotype. 

To address this hypothesis, we created nine tissue microarrays (TMA) from 233 treatment-naïve prostatectomy specimens from AA (118) and CA (115) patients. The patient characteristics and demographics are provided in Table 1. Serum PSA and Gleason scores were matched between the races to ensure that the tumor stage and grade do not contribute to differences in the expression of AR-SVs. The TMAs were stained with AR NTD and LBD-binding antibodies, and with an AR-V7 antibody. The staining intensity was quantified, and an H-score was provided for each specimen.

### 3.2. Treatment-Naïve Primary PCa Specimens Express AR-SVs

**Figure 3:**
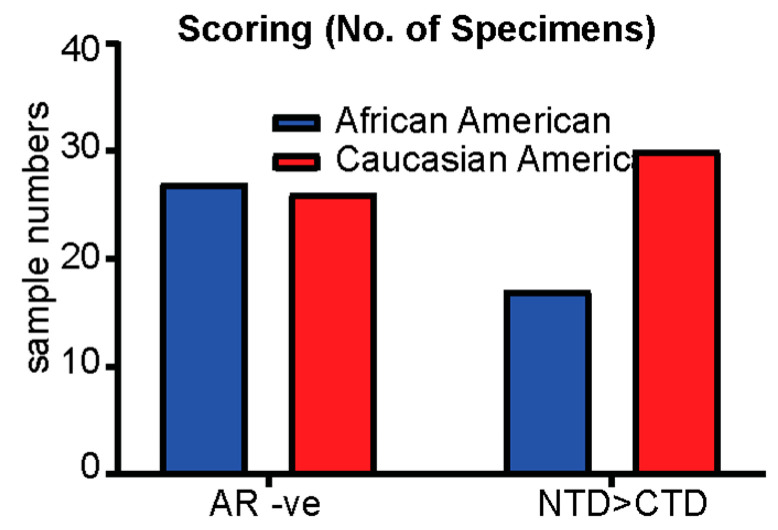
Expression of AR and AR-SVs. AR staining with antibodies binding to the N-terminus domain (NTD) and ligand-binding domain (LBD) was quantified and represented as H-score. The bars represent the number of specimens that did not stain with both the antibodies and the number of specimens that stained with NTD antibody at least 2.0-fold more than the LBD antibody. No statistical significance was observed between the groups.

**Figure 4:**
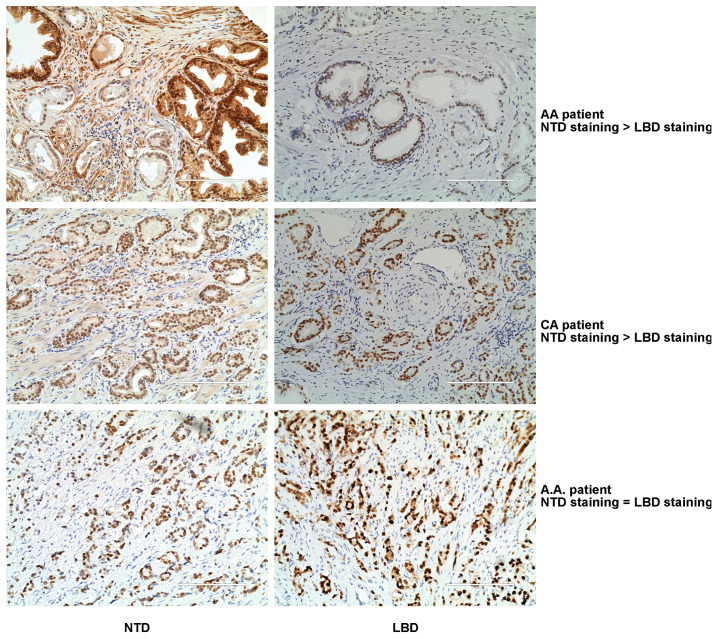
NTD and LBD antibody staining representative images from three patient specimens.

Out of the 221 evaluable specimens, 53 specimens (24%) did not stain for AR with either antibody. This suggests that these patients may not respond to any AR-targeted therapeutics and could potentially develop AR-negative neuroendocrine PCa. No racial differences were observed (27 vs. 26) (Figure 3). The pathological grade of AR-negative specimens was T2C or higher, with a Gleason score of 7 or higher. Only two patients, both AA men, stained positive for AR-V7. There were 47 patients with a rate of NTD staining 2-fold greater than that of LBD staining, corresponding to 21% of PCa specimens potentially expressing AR-SVs. This included 17 from AA men and 30 from CA men. No statistical significance between the races was identified. Representative IHC images are shown in Figure 4. Specimens (*n* = 6; 3 AA + 3 CA) were randomly selected, RNA was extracted, and real-time PCR was performed with TaqMan probes binding to the NTD and LBD. The results at the mRNA level confirmed the observation made with IHC.

## 4. Discussion

While most studies are performed to detect the AR-SVs focus on CRPCs, we focused on primary PCa with the assumption that expression of AR-SVs at an early stage might correlate with late-stage aggressive cancer. This study is potentially one of a few to take a distinct approach to identify AR-SVs in prostatectomy specimens with a ratio of N-terminus to C-terminus staining as a defining measure of AR-SV expression. An earlier study utilizing the same approach did not identify AR-SVs in primary PCa [37]. This approach in our patient cohort identified ~20% of primary PCa specimens expressing AR-SVs, including two AA patients with AR-V7 expression. AR-SVs could potentially be expressed in PCa specimens at higher rate than previously known. Considering that 20 AR-SVs [38] have been clinically identified, the proportion of AR-SVs in primary PCa and CRPC could be underestimated.

AR-V7 was detected in less than 1% of primary PCa specimens, while it was detected in over 75% of PCa specimens where the patients underwent androgen deprivation therapy (ADT) [19]. This number further increased in patients treated with abiraterone acetate or enzalutamide. Not many studies have comprehensively evaluated the expression of AR-SVs in treatment-naïve primary PCa specimens. At this time, more than 20 AR-SVs have been detected clinically and preclinically [38]; however, AR-V7 is the only AR-SV that can be reliably measured clinically. Since the NTD is highly conserved in almost all of the AR-SVs, our approach might provide a method to determine if there are AR-SVs expressed in PCa and CRPC specimens. Detecting the overall expression of AR-SVs in PCa specimens will help with the choice of treatment and will also provide an explanation for the failure to respond to treatments. With the advent of new AR NTD-targeting treatment approaches [33,34,35,36] comes the ability to detect AR-SVs early in the PCa occurrence, and using this approach suggests that these new AR NTD-targeting drugs might be beneficial to a broader spectrum of PCa patients. The IHC results were confirmed using real-time PCR with the limited availability of the specimens. Unfortunately, the specimens used in this manuscript are very old and enough tissues are not available to perform Western blot analysis.

Though we expected a racial disparity in the expression of AR-SVs, with AA patients expressing at a higher rate than the CA patients, we did not find any statistical difference between the two races in terms of AR-SV expression. Interestingly, the CA patients had a higher proportion of AR-SV-positive tumors. 

An earlier study with a small number of (*n* = 10) hormone-responsive bone metastasis specimens was performed to detect AR, AR-V7, AR-V1, and AR-v567es at mRNA levels. The study found that mRNA was detected in most of the primary tumors and metastasis, and this number increased in CRPC patient specimens [10]. Though that study evaluated the expression at the mRNA level, this study provides an independent validation for the expression of AR-SVs in early-stage PCa.

Strengths of the analysis include large number of AA and CA specimens with comparable ranges of age, PSA, and Gleason scores. The assumption that higher NTD staining relative to LBD was indicative of AR-SV expression was based on in vitro analyses with LNCaP and 22RV1 cell staining. However, there are limitations, including the potential for the masking of the antibody epitope in specimens that stained weakly at the LBD, hence providing a false positive interpretation that AR-SVs are expressed in primary PCa. Our results need to be confirmed using one of the sequencing methods such as probe-capture sequencing. Irrespective of these limitations, the study provides evidence that the expression of AR-SVs is not limited to CRPC, as previously thought, but could be expressed in primary PCa at a much higher rate and that the expression of AR-SVs in primary PCa might be underestimated clinically.

## Figures and Tables

**Table 1:** Patient demographics and specimen characteristics.

Characteristics	African American	Caucasian American
*n*	118	115
Age	59.65	62.12
BMI	29.02	28.05
PSA (ng/mL)	6.2 (*n* = 67)	4.7 (*n* = 90)
Gleason score (n)		
6	61	61
3 + 4	29	35
4 + 3	17	11
8	3	6
9	4	4
AR-V7	2	0
AR NTD antibody (+ve) n	56	67
AR NTD antibody (+ve) (intensity)	30	30
AR NTD antibody (−ve) *n*	47	48
AR LBD antibody (+ve) n	73	67
AR LBD antibody (+ve) (intensity)	30	30
AR LBD antibody (−ve) *n*	60	100
AR NTD and LBD antibody (−ve) *n*	27	26
Gleason score (NTD-H-score) mean		
6	34	25.5
3 + 4	39.7	32.9
4 + 3	15.6	42.3
8	1.7	8.3
9	20	50
Gleason score (CTD-H-score) mean		
6	59.4	52.2
3 + 4	103.8	68.2
4 + 3	65.9	70
8	8.3	10
9	62.5	103.3

## Data Availability

Data are stored in local hard drives. Since no sequencing data were published in this manuscript, no data was deposited in public database.
